# Increase of Suicide and Accidental Death After Hematopoietic Stem Cell Transplantation: A Cohort Study on Behalf of the Late Effects Working Party of the European Group for Blood and Marrow Transplantation (EBMT)

**DOI:** 10.1002/cncr.27987

**Published:** 2013-03-19

**Authors:** André Tichelli, Myriam Labopin, Alicia Rovó, Manuela Badoglio, Mutlu Arat, Maria Teresa van Lint, Anita Lawitschka, Carl Philipp Schwarze, Jakob Passweg, Gérard Socié

**Affiliations:** 1 Division of Hematology, University Hospital BaselBasel, Switzerland; 2 Department of Hematology–Bone Marrow Transplantation, Hospital Saint AntoineParis, France; 3 Florence Nightingale Sisli Hospital, Hematopoietic Stem Cell Transplantation UnitIstanbul, Turkey; 4 Ospedale San Martino, Department of Hematology IIGenova, Italy; 5 St. Anna Kinderspital, Stem Cell Transplant UnitVienna, Austria; 6 Paediatric Hematology and Endocrinology, University Children's HospitalTübingen, Germany; 7 Department of Hematology–Bone Marrow Transplantation, Hôpital St. Louis, ParisFrance

**Keywords:** hematopoietic stem cell transplantation, late effects, suicides, accidents, long-term, survivorship

## Abstract

**BACKGROUND:**

Relapse and transplant-related complications are leading causes of mortality after hematopoietic stem cell transplantation (HSCT). Suicides and accidents have not been studied in these patients. This study sought to determine whether there is an excess of suicide and accidental deaths after HSCT, and to determine risk factors.

**METHODS:**

The incidence of suicidal and accidental death in patients after undergoing HSCT, standardized mortality ratio (SMR), and absolute excess risk (AER) of suicide and accidental deaths was determined, compared with the general European population. A case-control analysis was done to define factors associated with suicide and accidental deaths. Data were derived from the European Group for Blood and Marrow Transplantation Registry, including 294,922 patients who underwent autologous or allogeneic HSCT from 1980 to 2009.

**RESULTS:**

The 10-year cumulative incidence of suicide and accidental deaths was 101.8 and 55.6 per 100,000 patients, respectively. SMR and AER of suicide after HSCT were 2.12 (*P* < .001) and 10.91, higher than in the European general population for 100,000 deaths, respectively. SMR and AER of accidental death were 1.23 (*P* < .05) and 2.54, respectively. In the case-control study, relapses were more frequent among patients who committed suicide after autologous HSCT (37% versus 18%; *P* < .0001). Chronic graft-versus-host disease was higher among patients who committed suicide after allogeneic HSCT (64% versus 37%; *P* = .001).

**CONCLUSIONS:**

There is an excess of deaths due to suicide and accidents in patients after undergoing HSCT as compared with the European general population. Relapse was associated with more suicide and accidental deaths after autologous HSCT, and chronic graft-versus-host disease was associated with more deaths by suicide after allogeneic HSCT. Cancer 2013;119:2012–2021. © 2013 American Cancer Society.

## INTRODUCTION

Hematopoietic stem cell transplantation (HSCT) is a treatment option for a variety of malignant and nonmalignant disorders.^1^–^3^ Overall survival has improved substantially over the last decade,^4^ but HSCT remains associated with considerable early and late treatment-related morbidity and mortality. When compared to a matched general population, mortality remains increased.^5^–^7^ Relapse of the primary disease and transplant-related mortality are the main causes of mortality after HSCT. With longer follow-up, other causes of death are observed, such as deaths from secondary malignancies, cardiovascular diseases, and other late organ dysfunctions.^8^,^9^ External causes of death, such as suicide and accident, are usually considered to be unrelated to the HSCT.

Suicide and accident are an important cause of death in a general population worldwide. In population-based epidemiological studies, cancer survivors have an elevated risk for committing suicide.^10^,^11^ Regarding accident-related deaths, there are no data for cancer patients. However, physical and psychological disabilities due to cancer and/or its treatment could intuitively lead to an excess of death from accidents. In patients treated with HSCT, the risk of suicide and accidents has never been systematically evaluated. Compared with patients who received recent cancer diagnoses, the situation is quite different, because patients who undergo HSCT have in most cases already been confronted with the diagnosis of malignancy, and have undergone intensive chemotherapy.

Why might patients who undergo HSCT be at higher risk of suicide? Depression, anxiety, and psychological dysfunction are common after HSCT.^12^ At some point during treatment or recovery, more than 20% of patients have symptoms consistent with clinical depression. Risk factors for depression after HSCT are a higher level of anxiety, decreased physical activity, less satisfaction in social activity before HSCT, and chronic graft-versus-host disease (GVHD) after transplantation.^13^ Depression has relevant repercussions on patient outcome: Persistent distress and depression can lead to poor return to function, and has been associated with increased mortality after transplantation.^14^ Furthermore, failure to meet the return to normal life after HSCT could be linked to depression and poorer psychological adjustment.^15^,^16^ In a general population, depression, anxiety disorders, and sleep disturbance are primary risk factors for suicidal ideation,^17^,^18^ and as a result, survivors of HSCT could be at increased risk for suicide.

Why might HSCT patients be at higher risk of accidents? Long-term survivors of HSCT report symptom burden including cognitive problems, fatigue, insomnia, musculoskeletal symptoms, emotional distress, anger, and depression.^19^ They may also have physical dysfunctions such as visual and auditory impairments, as well as cardiac or neurological problems.^9^ In an elderly general population, poor physical and mental condition, as well as the use of some medications, drugs, and alcohol are linked with reduced driving performance and increased crash rates.^20^,^21^ It is therefore reasonable to assume that physical impairment and psychological disabilities observed after transplantation may play a role in accident occurrence. In addition, some of the drugs needed in the posttransplant phase may decrease attention and interfere with the ability to adequately solve activities of daily living (causing for instance impaired driving capacity), and therefore be responsible for an excess of accidents after HSCT.

For these reasons, we expected an excess of deaths due to suicide and accidents in patients who were treated with HSCT. We therefore sought to estimate in a large cohort of patients from the European Group for Blood and Marrow Transplantation (EBMT) registry the risk of, and factors associated with, death from suicide and accidents among patients treated with HSCT, compared with the death rates of the European population in general.

## MATERIALS AND METHODS

### Database Population

This retrospective observational study was based on the mandatory minimum data set centers reported to the EBMT. The EBMT maintains a patient database known as the EBMT registry that goes back to the beginning of the 1970s and contains patient clinical data, including aspects of the diagnosis, first-line treatment, HSCT-associated procedures, complications, and outcome. Patients are reported exhaustively and followed up indefinitely; data are collected prospectively. According to the guidelines of the EBMT registry, it is the center's responsibility to ensure that the patient has provided consent before data is forwarded to the EBMT. As of March 2011, the EBMT database included 380,227 patients, from 661 transplant centers in 53 countries, who underwent autologous or allogeneic HSCT. We restricted the analysis to patients treated for hematological malignancy, solid tumors, marrow failure syndrome, and autoimmune diseases, who underwent transplantation between 1980 and 2009, excluding those with missing data on survival state, type of transplantation (autologous versus allogeneic), initial diagnosis, as well as patients who underwent transplantation for inborn errors. For patients who underwent transplantation more than once, only first the transplant was considered. Finally, 294,922 patients (77.6%) reported to the EBMT were included in this analysis. Causes of death were reported by transplant centers to the EBMT registry. We based the study on this source of data.

### Cohort Analysis

The primary endpoints were the cumulative incidence of death from suicide and accident, the deaths rates by suicide and accident, as well as the standardized mortality ratio (SMR) and the absolute excess risk (AER) of suicides and accidents compared with the general European population. Cause of death was defined as nonexternal (relapse-related deaths, transplant-related mortality, late transplant-related deaths such as late organ failure, secondary tumors) and external causes of deaths, which were defined as a death due to accident or suicide, according to the International Statistical Classification of Diseases and Related Health Problems, 10th Revision (ICD-10) from the World Health Organization.^22^ For this study, deaths by euthanasia or murder were not included in the group of external deaths. The cumulative incidence of death by suicide and accident after HSCT was calculated taking into consideration the competing risk of death due other causes.^23^ The time to risk was computed from the date of HSCT to the date of death or the date of last contact. Univariate analyses were performed using the Gray test. Factors considered were patient-related (sex, age), disease-related (diagnosis, disease status at HSCT), and type of transplant (autologous or allogeneic). Multivariate analysis was performed to calculate hazard ratio (HR) and their 95% confidence intervals (CI), adjusted for all covariates, using Cox proportional hazards regression model.

The statistics from the Eurostat, the statistical office of the European Union, which provides information at the European level including data from 15 representative European countries, were used to generate sex-specific death rates for suicide and accidents.^24^ These death rates were used to calculate expected number of cases for our cohort. The SMR was calculated by obtaining the ratio of the observed and expected number of cases. The 95% CIs were estimated using a method described by Haenszel. The AER of death by suicide and accident per 100,000 person-years of observation was calculated by subtracting the number of expected deaths from the observed, dividing by person-years of follow-up for the HSCT cohort multiplied by 100,000.

### Case-Control Analysis

We used a nested case-control approach to identify prognostic factors related to disease and HSCT characteristics on risk of death by suicide and accidents after HSCT. For each case of suicide and accident, 3 controls were selected within the cohort according to the following matching criteria: type of HSCT, age at HSCT by decade, patient sex, year of transplantation, and length of follow-up (control's follow-up was equivalent to, or exceeded that, of the index case). Prognostic factor analyses were performed separately for allogeneic and autologous HSCT. Cases and controls were compared with respect to disease, disease stage at HSCT (standard versus high risk), type of donor, and relapse (for controls, relapse occurring at equivalent time to or before death of the index case). For the allogeneic HSCT cohort, cases and controls were further compared with respect to type of conditioning (myeloablative versus reduced intensity), total body irradiationTBI for patients treated with myeloablative conditioning, and GVHD (for controls, GVHD occurring at equivalent time to or before death of the index case). A conditional logistic regression was used to compare the 2 groups and to estimate ORs. All *P* values are 2-sided with type I error rate fixed at .05. Statistical analyses were performed with SPSS Statistic 19 (IBM Corporation) and R 2.13.2 software packages (R Development Core Team, Vienna, Austria).

## RESULTS

### Cohort Analysis

From the 294,922 patients included in the cohort study, 108,951 patients (36.9%) had received allogeneic and 185,971 patients (63.1%) had received autologous HSCT. From this cohort, 57% were males. The median age at HSCT was 34.2 years at allogeneic and 48.4 years at autologous HSCT. Most patients (96%) underwent transplantation for hematological malignancies; 53% were considered as standard and 47% high-risk at time of HSCT. More than 60% of the patients underwent transplantation after 2000; the median follow-up of patients alive at last follow-up was 2.34 years (range, 1 day to 31 years).

#### Causes of Death After HSCT

In total, 116,149 patients (39.4%) died. The main causes of death were relapse in 61,605 patients (57.5%), transplant-related mortality in 38,600 patients (36%), and late transplant-related causes of death in 6671 patients (6.5%). There were 314 (0.27%) external causes of death, 189 suicides, and 125 deaths by accident.

**Figure 1:**
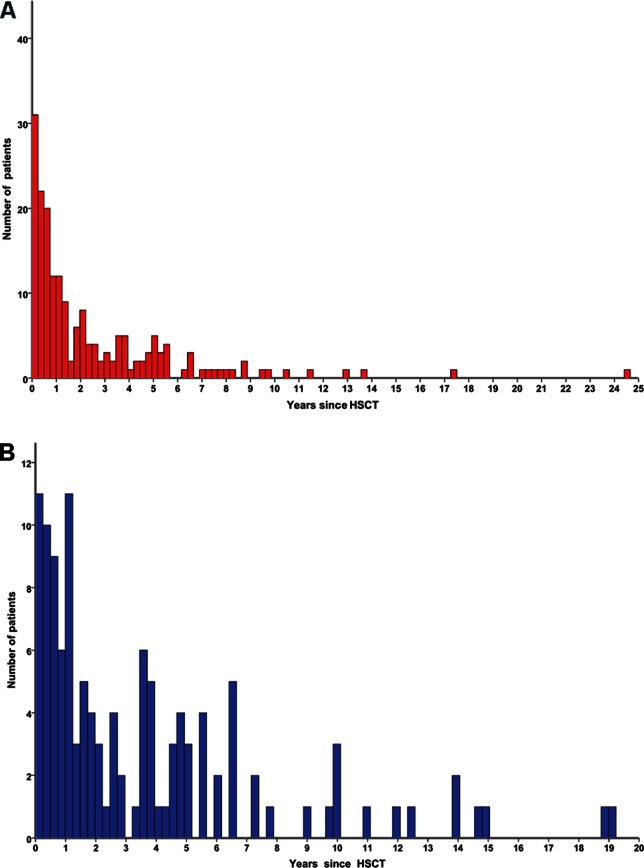
Graphs show the (A) time interval between hematopoietic stem cell transplantation (HSCT) and death by suicide, and (B) time interval between HSCT and death by accident.

Of the 189 patients with death from suicide, 140 (74%) were males, 74 (39%) received allogeneic HSCT, and 115 (61%) received autologous HSCT. The median age at HSCT and at suicide was 45 years (range, 6-73 years) and 46 years (range, 16-73 years), respectively. The median time interval between HSCT and suicide was 13 months (range, 1-292 months). Death due to suicide after HSCT occurred within the first year in 86 patients (46%), and during the second year in 29 patients (15%). In 32 patients (17%), suicide occurred after 5 years (Fig. 1A).

Of the 125 patients with death from accident, 92 (74%) were males, 48 (38%) received allogeneic HSCT, and 77 (62%) received autologous HSCT. The median age at HSCT and at time of the accidental death was 41 years (range, 1-69 years) and 45 years (range, 2-71 years), respectively. The median time interval between HSCT and accident was 13 months (range, 1-292 months). Death due to accident occurred within the first year in 40 patients (32%) and during the second year in 22 patients (18%). In 30 patients (24%), accidents occurred after 5 years (Fig. 1B). Fifty-one patients died of traffic accident, 9 of fall, 6 of drowning, 2 of farm accident, and 2 of fire. In 55 cases (44%), the type of accident was not further specified.

#### Cumulative Incidence and Risk Factors

**Figure 2:**
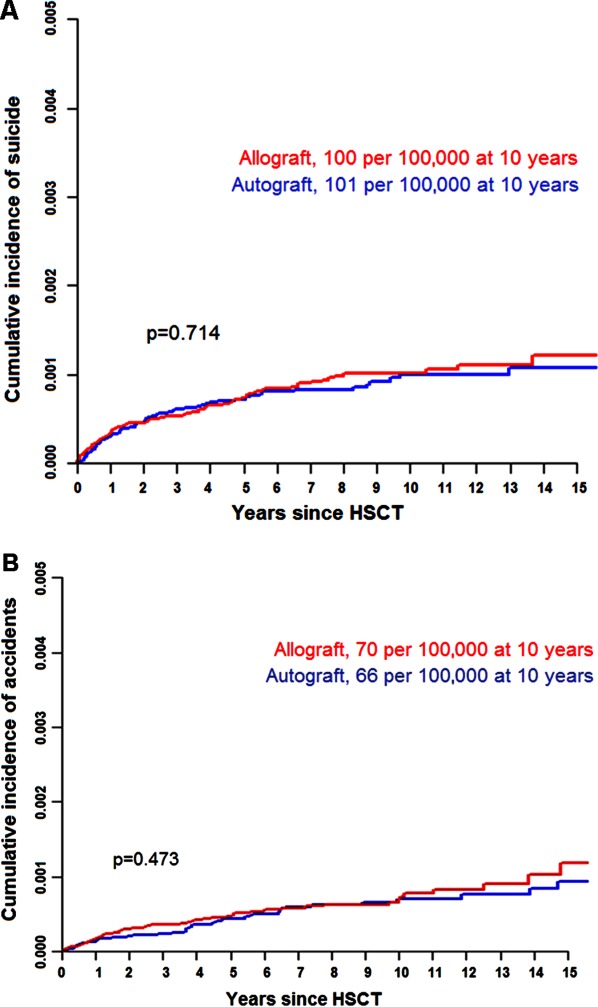
Graphs show (A) cumulative incidence (CI) of deaths by suicide after allogeneic and autologous hematopoietic stem cell transplantation (HSCT): For allograft, CI is 100 per 100,000 at 10 years. For autograft, CI is 101 per 100,000 at 10 years. (B) CI of deaths by accident after allogeneic and autologous HSCT are shown. For allograft, CI is 70 per 100,000 at 10 years. For autograft, CI is 66 per 100,000 at 10 years.

The cumulative incidence of suicide death at 5 and 10 years was 72.9 and 101.8 per 100,000 patients, respectively. It was 72 and 100 per 100,000 patients for allogeneic HSCT, and 73 and 101 per 100,000 patients for autologous HSCT (*P* = .71), respectively (Fig. 2A). The cumulative incidence of accidental death at 5 and 10 years after HSCT was 47.4 and 55.6 per 100,000 patients, respectively. It was 44 and 70 per 100,000 patients for allogeneic HSCT, and 49 and 66 per 100,000 for autologous HSCT (*P* = .47) (Fig. 2B).

Results of univariate analysis are summarized in Table 1. By multivariate analysis, suicide rate after HSCT was lower in females compared with males (OR = 0.43, 95% CI = 0.30-0.61; *P* = .0001), and significantly associated with age (*P* = .001) (Table 2). After HSCT, accidental deaths were less frequent in females (OR = 0.45; 95% CI = 0.30-0.68; *P* = .0001) (Table 2).

**TABLE 1:** Cumulative Incidence of Death per 100,000 Patients at 5 Years Due to Suicide and Accident After HSCT

	Suicide Death		Accident Death	
		
Characteristic	5-y per 100,000	*P*	5-y per 100,000	*P*
All patients	72.9	–	47.4	–
Type of HSCT				
Allogeneic	72		44	
Autologous	73	.71	49	.47
Sex				
Male	91		61	
Female	50	<.0001	30	.0002
Age at HSCT				
1-14	7		24	
15-24	54		43	
25-34	83		64	
35-44	91	.0002	36	.42
45-54	107		50	
55-64	60		66	
>65	101		38	
Primary disease				
AL, MDS, MPN	73		46	
CML	85	.18	14	.02
Lymphoma, myeloma, CLL	80		57	
Solid tumor	47		26	
Nonmalignant diseases	38		74	
Disease status at HSCT				
Standard risk	79		55	
High risk	64	.18	42	.24
Calendar year of HSCT				
1980-1989	81		38	
1990-1994	51		23	
1995-1999	65	.2	36	.13
2000-2004	82		63	

Abbreviations: AL, acute leukemia; CLL, chronic lymphocytic leukemia; CML, chronic myelocytic leukemia; HSCT, hematopoietic stem cell transplantation; MDS, myelodysplastic syndrome; MPN, myeloproliferative neoplasm.

**TABLE 2:** Multivariate Analysis for Risk Factors for Suicidal and Accidental Deaths: Cohort Study

	Suicidal Deaths				Accidental Deaths			
		
			95% CI				95% CI	
						
Characteristic	*P*	HR	inf	sup	*P*	HR	inf	sup
**Autologous versus allogeneic**	.93	0.98	0.62	1.54	.84	0.95	0.55	1.62
**Sex**								
Male (reference)		1				1		
Female	<.0001	0.43	0.30	0.61	<.0001	0.45	0.30	0.68
**Age at HSCT, y**	.001				.23			
1-14 (reference)		1				1		
15-24	.06	2.54	0.97	6.68	.04	2.43	1.04	5.70
25-34	.001	4.47	1.80	11.09	.009	3.07	1.32	7.16
35-44	.000	5.77	2.37	14.05	.06	2.34	0.98	5.61
45-54	.000	6.48	2.65	15.81	.02	2.77	1.16	6.61
55-64	.001	4.84	1.90	12.34	.009	3.30	1.34	8.11
>65	.002	5.83	1.90	17.91	.06	3.21	0.96	10.76
**Primary disease**	.764				.13			
AL, MDS, MPN (reference)		1				1		
CML	.50	0.82	0.47	1.44	.02	0.33	0.13	0.86
Lymphoma, CLL, myeloma	.21	0.73	0.45	1.20	.67	0.88	0.49	1.58
Solid tumors	.55	0.79	0.36	1.72	.31	0.57	0.20	1.67
Nonmalignant disorders	.72	0.84	0.33	2.15	.49	1.32	0.60	2.91
**Risk status at HSCT**								
Standard risk (reference)								
High risk	.77	1.05	0.74	1.51	.63	0.90	0.58	1.39
**Calendar year of HSCT**	.35				.29			
1980-1989 (reference)		1				1		
1990-1994	.04	0.48	0.24	0.97	.11	0.54	0.25	1.15
1995-1999	.15	0.65	0.36	1.17	.19	0.64	0.32	1.26
2000-2004	.22	0.69	0.38	1.25	.78	0.91	0.46	1.79
2005-2009	.29	0.71	0.37	1.34	.36	0.70	0.32	1.51

Abbreviations: AL, acute leukemia; CI, confidence interval; CLL, chronic lymphocytic leukemia; CML, chronic myeloid leukemia; HR, hazard ratio; inf, inferior; MDS, myelodysplastic syndrome; MPN, myeloproliferative neoplasm; sup, superior.

#### Suicide Death Rates

The suicide death rate was 20.7 per 100,000 person-years after HSCT. The suicide death rates were 27.4 and 14.0 per 100,000 person-years for males and females, respectively. The total expected suicide death rates according to Eurostat are 9.2 per 100,000 person-years (males, 14.0; females, 4.4) (Table 3). Thus, the SMR and the AER after HSCT were 2.12 (95% CI = 1.83-2.45; *P* < .001), and 10.91 higher than in the European for 100,000 deaths, respectively. Standardized rates were higher both in males (SMR, 1.96; 95% CI = 1.65-2.31; *P* < .001; AER, 13.4) and in females (SMR, 2.77; 95% CI = 2.05-3.67; *P* = .001; AER, 7.81) (Table 3).

**TABLE 3:** Death Rates per 100,000 Person-Years, Expected Death Rates, Standardized Mortality Ratio, and Absolute Excess Ratio per 100,000 for Suicides and Accidents, Including All Patients After Hematopoietic Stem Cell Transplantation and Stratified by Sex

Population	Person-Years	Suicide Rates per 100,000	Expected Suicide Rates^a^	O/E	SMR (95%CI)	AER (for 100,000)
**Deaths by suicide**						
Overall	915,183	20.7	9.2	189/89	2.12 (1.83-2.45)	10.91
Male	510,700	27.4	14.0	140/71	1.96 (1.65-2.31)	13.41
Female	401,401	12.2	4.4	49/18	2.77 (2.05-3.67)	7.81
**Deaths by accident**						
Overall	915,183	13.7	10.5	125/102	1.23 (1.02-1.46)	2.54
Male	510,700	18.0	16.3	92/83	1.11 (0.89-1.36)	0.98
Female	401,401	8.2	4.6	33/18	1.83 (1.26-2.57)	1.64

a Expected death rates from Eurostat.

Abbreviations: AER, absolute excess ratio for 100,000; O/E, observed number versus expected number of deaths; SMR, standardized mortality ratio.

**Figure 3:**
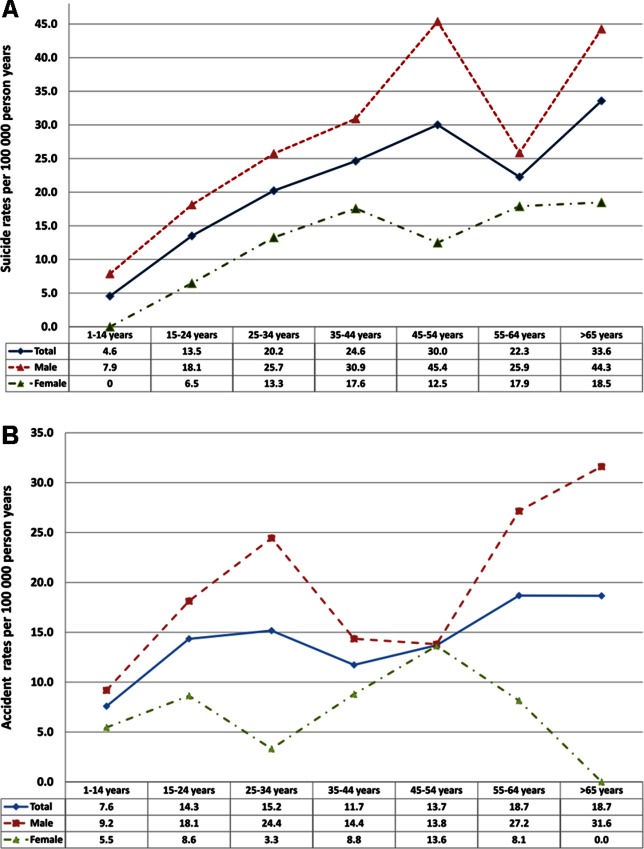
Graphs show (A) suicidal death rates per 100,000 person-years, by sex and age at hematopoietic stem cell transplantation, and (B) accidental death rates per 100,000 person-years, by sex and age at hematopoietic stem cell transplantation.

We further compared the suicide death rates among age groups at time of HSCT (Fig. 3A). As in the general population, there was a continuous increase in the death rates in the whole cohort, as well as in males and in females when they were analyzed separately. In the whole cohort, it increased from 5.0 deaths per 100,000 person-years for patients aged 1 to 14 years at time of HSCT up to 34 deaths per 100,000 person-years in patients older than 65 years.

#### Accidental Death Rates

Accidental death rates after HSCT were 13.7 per 100,000 person-years (males, 18.0; females, 8.2). The expected accident death rates in the European general population, according to Eurostat statistics, are 10.5 per 100,000 person-years (males, 16.3; females, 4.9). Thus, the SMR and AER were 1.23 (95% CI = 1.02-1.46; *P* = .05) and 2.54, respectively. Standardized death from accident was significantly increased in females (SMR, 1.83; 95% CI = 1.26-2.57; *P* = .01) but not in males (SMR, 1.11; 95% CI = 0.89-1.36) (Table 3).

We also compared accident death rates by age groups at the time of HSCT (Fig. 3B). These death rates were lowest in patients who underwent transplantation before the age of 15 years (7.6 deaths) and highest in patients older than 55 years at time of HSCT (18.7 deaths). For patients aged between 15 and 55 years at the time of HSCT, however, there was a constant death rate between 12 and 15 accident deaths per 100,000 person-years.

### Case-Control Study

The 189 patients with suicide (cases) were matched with 560 patients who did not die from suicide (controls). Pretransplant factors, relapse rate, GVHD incidence, type of conditioning, and donor type are summarized for cases and controls in Table 4. Relapses were significantly more frequent among patients who died from suicide (52 of 189 patients [28%] versus 81 of 560 patients [15%]; *P* = .02). This difference was observed in patients who underwent autologous HSCT (37% [42 of 115 patients] versus 18% [60 of 342 patients]; *P* < .0001), but not in those treated with allogeneic HSCT (14% [10 of 71 patients] versus 10% [21 of 212 patients]; *P* = .33). The prevalence of chronic GVHD was significantly higher among patients who committed suicides after allogeneic HSCT and who survived at least 100 days, as compared with controls (64% [29 of 45 patients] versus 37% [62 of 168 patients]; *P* = .001).

**TABLE 4:** Nested Case-Control Study for the Risk of Suicide and Accidentsa^a^

	Suicide Death			Accidental Death		
		
Characteristics	Cases (n = 187)	Controls (n = 560)	*P*	Cases (n = 125)	Controls (n = 372)	*P*
**All patients treated with HSCT**						
High risk at HSCT	76/174 (44%)	233/520 (45%)	.8	47/120 (39%)	138/357 (39%)	.92
Relapse	52/186 (28%)	81/554 (15%)	<.0001	26/124 (21%)	45/368 (12%)	.02
**Patients treated with allogeneic HSCT**						
Family donor	49/71 (69%)	143/212 (67%)	.81	38/45 (84%)	111/139 (80%)	.5
Reduced intensity conditioning	12/68 (18%)	36/201 (18%)	.96	8/47 (17%)	27/140 (19%)	.73
Relapse	10/71 (14%)	21/212 (10%)	.33	5/48 (10%)	12/140 (9%)	.7
Acute GVHD	28/69 (41%)	62/194 (32%)	.2	17/45 (38%)	38/132 (29%)	.26
Chronic GVHD^b^	29/45 (64%)	62/168 (37%)	.001	14/32 (44%)	39/104 (38%)	.53
TBI in MAC	34/55 (62%)	106/162 (65%)	.63	26/36 (72%)	65/107 (61%)	.22
**Patients treated with autologous HSCT**						
High risk at HSCT	65/110 (59%)	179/320 (56%)	.57	36/74 (49%)	105/220 (52%)	.59
Relapse	42/115 (37%)	60/342 (18%)	<.0001	21/76 (28%)	33/228 (14%)	.009
TBI	17/104 (16%)	43/314 (14%)	.5	6/71 (8%)	23/214 (11%)	.58

a Case-control matching criteria included type of HSCT, age at HSCT by decade, sex of the patients, year of transplantation, and length of follow-up.

b Including only patients surviving at least 100 days after HSCT.

Abbreviations: GVHD, graft-versus-host disease; HSCT, hematopoietic stem cell transplantation; MAC, myeloablative conditioning; TBI, total body irradiation.

The 125 patients with accidents (cases) were matched with 372 patients who did not die from accidents (controls). Relapse in patients treated with autologous HSCT was significantly more frequent in patients who died from accidents than in controls (28% [21 of 76 patients] versus 14% [33 of 228 patients]; *P* = .009). This was not the case for patients treated with allogeneic HSCT.

Conditional logistic regression analysis was performed in patients treated with allogeneic and autologous HSCT. Patients who experienced chronic GVHD after allogeneic HSCT had a 2.9-fold (OR = 2.91; 95% CI = 1.16-7.23; *P* = .002) and patients relapsing after autologous HSCT had a 4.5-fold (OR = 4.48; 95% CI = 2.3-8.76; *P* < .0001) increased risk of dying from suicide (Table 5).

**TABLE 5:** Nested Case-Control Study: Conditional Logistic Regression Analyses for the Risk of Suicide and Accidents

	Suicide Death			Accident Death		
		
Parameter	Odds Ratio	95% CI	*P*	Odds Ratio	95% CI	*P*
**Allogeneic HSCT**						
Chronic GVHD						
No GVHD	1		.02			
With GVHD	2.91	1.16-7.23				
Risk group at HSCT						
Standard risk	1		.08			
High risk	0.36	0.11-1.12				
Relapse						
None	1		.08			
Yes	3	0.88-10.25				
**Autologous HSCT**						
Relapse						
None	1		<.0001	1		.02
Yes	4.48	2.3-8.76		2.36	1.16-4.81	
Risk group at HSCT						
Standard risk	1		.81	1		.32
High risk	0.94	0.57-1.56		0.73	0.39-1.36	
TBI for conditioning						
None	1		.45	1		.48
Yes	1.32	0.64-2.71		0.66	0.20-2.11	

Abbreviations: CI, confidence interval; GVHD, graft-versus-host disease; HSCT, hematopoietic stem cell transplantation; TBI, total body irradiation.

## DISCUSSION

This study, which included a large cohort of European patients, demonstrates for the first time that there is an excess of deaths due to suicide and accident after HSCT as compared with the European general population. The suicide death rate of the patients who underwent transplantation exceeded that of the European general population by a factor of 2, and there is a 10-fold increase of the absolute excess risk. SMR and AER were also increased for death from accident, a difference mainly observed in female patients. The overall risk factors for death related to suicide were male sex and older age. Despite similar cumulative incidences between autologous and allogeneic HSCT, factors linked to suicides and accidents appeared different; relapse was associated with more deaths by suicide and accident after autologous HSCT and chronic GVHD, with more deaths by suicide after allogeneic HSCT.

In the general population, risk groups for suicide include mainly male sex, older age, unemployment, drug abuse, mental illness, and depression. However, it now becomes evident that chronic physical illness is a relevant risk factor.^25^–^27^ One in 10 suicides is among people with a physical illness,^28^ and patients with cancer have nearly twice the expected incidence of suicide.^10^,^11^,^29^,^30^ Furthermore, one-third of outpatients with cancer have suicidal ideation.^31^ A large cohort study of Swedish patients who recently received a cancer diagnosis found these individuals were at increased risk of suicide death, as compared with cancer-free persons.^32^ Following solid-organ transplantation, depression appears to be one of the most common psychiatric disorders.^33^ Posttransplant depressive disorders occur between 5% and 25% in patients, and suicide rate after renal transplantation may be higher than expected in the general population.^33^,^34^

There is a paucity of data on suicide death after HSCT.^35^ In our study, the difference in risk elevation for suicide observed between allogeneic and autologous HSCT, ie, chronic GVHD after allogeneic and relapse after autologous transplantation, is conceivable. Serious GVHD leads to disability, chronic illness, and reduced patient-reported quality of life, and may therefore explain the higher risk of suicide in patients treated with allogeneic HSCT.^36^,^37^ In contrast, relapse of the primary disease is the main concern after autologous HSCT. Cancer patients with poor survival expectation and poor prognosis facing the possibility of palliative care are at highest risk of suicide.^32^,^38^,^39^ In a large study including 121,533 cancer patients, cancer recurrence shortly after treatment completion was postulated to be a trigger for suicide.^40^ In our study, the excess of suicide after relapse can be explained by the perception of a desperate issue after having failed a “last-chance” treatment. Cancer patients treated with HSCT probably have an additional risk for suicide death. Indeed, in cancer patients, this risk is highest immediately after diagnosis of the cancer.^40^ At the time of transplantation, most patients have already been confronted with the diagnosis of malignancy and have received intensive chemotherapy. Therefore, they have overcome the main period of risk for suicide death due to the cancer itself. Nevertheless, to determine if HSCT patients are at additional risk for suicide, a study comparing patients with a similar malignant disease treated with and without HSCT should be performed.

In this study, relapse was also related with higher risk of accidental death after autologous HSCT. Several reasons could explain the excess of deaths by accidents after HSCT. Accidents are associated with psychological impairments, anxiety, and life events.^41^ No risk factors for accidents have been found with allogeneic HSCT. Accidents can be attributed to inattention, poor sight or hearing, slow reactions, and other medical issues. Moreover, the close relationship between relapse and accidental death after autologous transplantation points to the possibility that some of the accidents may be intentional, and therefore represent a hidden suicide.^42^ Therefore, physical and psychological distress appearing after HSCT may be involved in the excess of accidents. In long-term survivors after allogeneic HSCT, late effects such as cataract, cardiovascular complications, neurological complications, as well as decreased cognitive functioning may occur,^43^–^51^ and be incriminated for the increased risk of accidental death.

The major strength of our study is the large-scale population-based cohort with essentially complete ascertainment of transplants and fatal outcomes in Europe. The cohort was large enough to provide reliable data on death rates by suicide and accidents. The case-control analysis allows for an evaluation of transplant-related risk factors. To the best of our knowledge, this is the first study to evaluate external cause of death on a large-scale cohort.

There are limitations to this study, mainly due to the retrospective design and the absence of data on specific nonfatal late complications after HSCT that could have indirect repercussions on accidental death, such as cardiovascular complications, neurological problems, decreased cognitive functioning, and visual or hearing acuity. Second, a potential bias for the validity of our findings could be the diverse way how suicide and accidental deaths are ascertained in the different European countries. Third, although suicide and accidents are at higher risk in patients who undergo transplantation, it remains unclear what makes the difference from other patients with similar medical risk, such as those with chronic illness and severe pain,^25^,^26^ end-stage renal disease,^27^ human immunodeficiency virus positivity,^52^ congestive heart failure, or chronic obstructive lung disease.^53^ Finally, we cannot rule out the possibility that there is an underestimation of deaths due to suicide or accidents. The source of the causes of death came from the EBMT registry, which is based on center reporting and not on certificate of death. However, this restriction would not affect the validity of our findings. We suggest that our conclusions are generalizable to patients treated with HSCT.

This is the first study to observe an increased risk for death due to suicide and accidents after HSCT, and to identify patients at risk. Relapse after autologous HSCT was associated with more suicides and accidents, and chronic GVHD after allogeneic HSCT with an excess of suicides. The transplant community and the health care providers should be informed about the possibility that a person after HSCT may be suicidal or be exposed to an accident. However, there are still open questions that need to be addressed in further studies. It is conceivable that in addition to chronic GVHD or relapse, other relevant risk factors such as deterioration of quality of life, adverse social issues, or specific physical and mental limitations could be responsible for the increased suicide or accidental death rate in patients treated with HSCT. Such a type of data was not available in this large registry, but perhaps next-generation studies could assist in defining patients at risk. Furthermore, suicide and accidental death cannot be ignored in data analysis on transplant outcome. These deaths have to be classified as transplant-related or external cause of death.

## FUNDING SOURCES

No specific funding was disclosed.

**CONFLICT OF INTEREST DISCLOSURE**

The authors made no disclosure.
